# Friction in Total Hip Joint Prosthesis Measured *In Vivo* during Walking

**DOI:** 10.1371/journal.pone.0078373

**Published:** 2013-11-08

**Authors:** Philipp Damm, Joern Dymke, Robert Ackermann, Alwina Bender, Friedmar Graichen, Andreas Halder, Alexander Beier, Georg Bergmann

**Affiliations:** 1 Julius Wolff Institute, Charité – Universitaetsmedizin Berlin, Berlin, Germany; 2 Klinik für Endoprothetik, Sana Kliniken Sommerfeld, Sommerfeld, Germany; Delft University of Technology (TUDelft), The Netherlands

## Abstract

Friction-induced moments and subsequent cup loosening can be the reason for total hip joint replacement failure. The aim of this study was to measure the *in vivo* contact forces and friction moments during walking. Instrumented hip implants with Al_2_O_3_ ceramic head and an XPE inlay were used. *In vivo* measurements were taken 3 months post operatively in 8 subjects. The coefficient of friction was calculated in 3D throughout the whole gait cycle, and average values of the friction-induced power dissipation in the joint were determined. On average, peak contact forces of 248% of the bodyweight and peak friction moments of 0.26% bodyweight times meter were determined. However, contact forces and friction moments varied greatly between individuals. The friction moment increased during the extension phase of the joint. The average coefficient of friction also increased during this period, from 0.04 (0.03 to 0.06) at contralateral toe off to 0.06 (0.04 to 0.08) at contralateral heel strike. During the flexion phase, the coefficient of friction increased further to 0.14 (0.09 to 0.23) at toe off. The average friction-induced power throughout the whole gait cycle was 2.3 W (1.4 W to 3.8 W). Although more parameters than only the synovia determine the friction, the wide ranges of friction coefficients and power dissipation indicate that the lubricating properties of synovia are individually very different. However, such differences may also exist in natural joints and may influence the progression of arthrosis. Furthermore, subjects with very high power dissipation may be at risk of thermally induced implant loosening. The large increase of the friction coefficient during each step could be caused by the synovia being squeezed out under load.

## Introduction

In 20% to 40% of all cases [1], polyethylene wear and aseptic loosening are the most frequent reasons for revisions of total hip joint replacements (THR). Both factors are related to friction in the joint. Cup loosening has been reported to be the only cause in 30% to 62% of revisions [2], [3]. Subjects, who obtained a THR are becoming increasingly younger and are, therefore, more active and athletic [4], [5]. However, higher activity levels produce more wear and more strenuous activities cause higher friction moments. This will increase the risk of implant loosening [6], [7]. These facts indicate that reduction of friction between head and cup is critical for further improvement of THR.

Several *in vivo* studies have been performed to investigate the loads acting in hip implants during different activities [8], [9]. These studies have shown that the contact force during normal walking falls in a range between 240 and 480% of the bodyweight (BW). However, *in vivo* measurements of friction in hip endoprostheses have not been reported previously.

One *in vivo* study *indirectly* assessed friction in the joint by measuring the implant temperature during an hour of walking [10], [11]. Its increase is mainly related to the friction-induced power generated in the implant. A peak temperature of 43.1°C was measured in 1 subject, a level at which bone tissue may already be impaired [12], especially when this temperature occurs repeatedly. Therefore, it cannot be excluded that friction and increased implant temperatures may be underestimated risk factors for the long-term stability of THR.

To determine the friction in total hip joint prosthesis, *in vitro* simulator studies were performed [13], [14]. To evaluate the friction between two sliding partners, the coefficient of friction µ was used. For the tribological pairing Al_2_O_3_/UHMWPE µ ranges depended on the lubricant [13], ranging from 0.044 (distilled water), to 0.054 (bovine serum), and 0.089 (saline). The coefficient increased dramatically up to values of 0.14 when the conditions changed from lubricated to dry [15].

However, most of the simulator tests load the joint only in the flexion-extension plane and use load patterns which may not be realistic [16]. Newer studies investigated friction under more realistic conditions, simulating *in vivo* measured gait data [17]. Varying parameters for friction were investigated, for example, different material combinations for implant head and cup [18], and various lubricant regimes [17], [19]–[22]. These simulator studies were performed under very different test conditions, such as deviating patterns of joint loading and movement or by using different lubricants. Nevertheless, it was shown that friction in THR is mainly influenced by the material of the sliding partners and the lubrication regime.

The aim of our study was to determine the *in vivo* contact forces in hip implants during walking, plus the moments caused by friction, and derive the coefficient of friction from these data. These data will help to understand the *in vivo* lubrication conditions and allow validating, potentially improving the conditions applied in joint simulators.

## Methods

### Ethic statement

The study was approved by the Charité Ethics committee (EA2/057/09) and registered at the ‘German Clinical Trials Register’ (DRKS00000563). All patients gave written informed consent prior to participating in this study.

### Subjects and measurements

Eight subjects with instrumented hip joint prostheses (Table 1) participate in this study. Measurements were taken 3 months postoperatively (pOP) during level walking at a self-selected walking speed. Selected trials of each investigated subject are also shown and can be downloaded at the public data base www.OrthoLoad.com.

**Table 1:** Patients investigated.

			Body	Gait	Mean Gliding Speed
Patient	Age	Gender	weight	Velocity	Extension | Flexion
	[years]		[N]	[m/s]	[m/s]
**H1**	56	m	754	1.0	0.02 | 0.04
**H2**	62	m	755	1.0	0.03 | 0.05
**H3**	60	m	880	0.8	0.02 | 0.06
**H4**	50	m	783	1.0	0.03 | 0.06
**H5**	62	f	853	0.9	0.02 | 0.08
**H6**	69	m	832	1.1	0.03 | 0.05
**H7**	53	m	899	1.1	0.03 | 0.06
**H8**	56	m	779	1.1	0.03 | 0.06
***Average***	*59*	*-*	*821*	*1.0*	*0.03 | 0.06*

### Measuring equipment

Joint forces and friction moments were measured *in vivo* with instrumented hip implants. The prosthesis (CTW, Merete Medical, Berlin, Germany) has a titanium stem, a 32 mm Al_2_O_3_ ceramic head (BIOLOX forte, CeramTec GmbH, Plochingen, Germany) and an XPE inlay (Durasul, Zimmer GmbH, Winterthur, Switzerland). A telemetry circuit, 6 strain gauges, and a secondary induction coil are placed in the hollow neck, which is closed by welding. A detailed description of the instrumented implant was published previously [23]. A coil around the hip joint inductively powers the inner electronics. The strain gauge signals are transferred via an antenna in the implant head to the external receiver. These signals and the subject's movements are recorded simultaneously on videotape. The external measurement system has previously been described in detail [24], [25].

**Figure 1:**
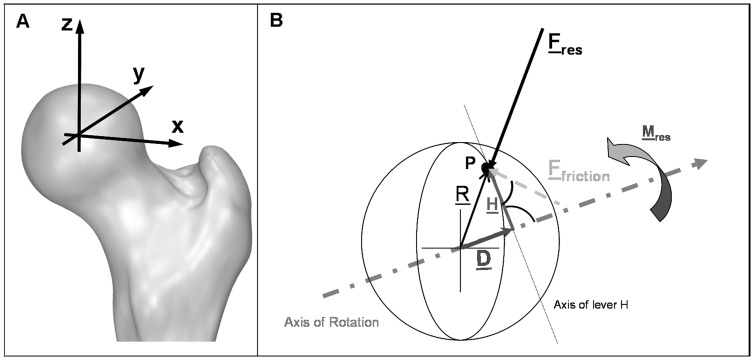
Coordinate system and vectors for calculation of the coefficient of friction µ. Right joint, posterior view.

From the 6 strain gauge signals, the 3 force and 3 moment components acting on the implant head are calculated [26] with an accuracy of 1–2%. The femur-based coordinate system [27] is fixed in the center of the head of a right sided implant, but is defined relative to the bone. Data from left implants were mirrored to the right side. The positive force components F_x_, F_y_, and F_z_ act in lateral, anterior, and superior directions, respectively (Figure 1A). From the 3 force components the resultant contact force F_res_ is calculated. The measured friction moments M_x_, M_y_, and M_z_, act clockwise around the positive axes. The 3 moment components deliver the resultant friction moment M_res_. Positive/negative moments M_x_ are caused by extension/flexion of the joint. Positive/negative moments M_y_ act during abduction/adduction. Positive/negative moments M_z_ are caused by external/internal rotation.

### Data evaluation

All forces are reported as percent of bodyweight (% BW) and the friction moments as % BWm. Average force-time patterns from 32–96 steps were calculated for each subject. The employed ‘time warping’ method [28] weighted the congruency of high forces more than that of lower ones. First the period times of all the included steps are normalized. The single time scales were then distorted in such a way that the squared differences between all deformed curves, summed over the whole cycle time, were smallest. Finally, an arithmetically averaged load-time pattern was calculated from all the deformed curves. Using these algorithms, an average time course was first calculated from the time patterns of the resultant joint forces F_res_. The obtained time deformations of the single trials were then transferred to the corresponding force and moment components before averaging them, too. The resultant friction moment M_res_ was calculated from an average of its components.

This procedure was first applied on all trials of the single subjects, leading to ‘individual’ averages. These averages from all subjects were then combined to a ‘general’ average, which describes the loads acting in an ‘average’ subject.

In some cases, peak values were not taken from the averaged time courses, but instead, the numerical peak values of the *single* trials were averaged, first individually and then inter-individually. Extreme values of the averaged load-time patterns can slightly deviate from these numerically averaged numbers. These values of the average subject were statistically analysed (p≤0.05; Wilcoxon).

### Coefficient of friction

The coefficient of friction µ between the head and the cup was calculated by a 3D approach (Figure 1B). Joint movement takes place in a plane perpendicular to the axis of the vector M_res_. This axis is not perpendicular to the axis of vector F_res_. The vector of the friction force F_friction_ acts perpendicular to F_res_ at point P on the head. H is the vector of the lever arm between F_Friction_ and the axis of M_res_ and is perpendicular to both. D is a vector in direction of M_res_. R = 16 mm is the radius vector to point P.

Assuming a Coulomb friction between the head and the cup, the friction force acting on the head is
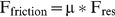
(1)


The moment determined by the force F_friction_ and its lever arm H has to counteract M_res_. Because F_friction_ and H are perpendicular to each other, this delivers the scalar equation
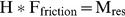
(2)


From the combination of (1) and (2), the following equation can be derived:
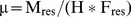
(3)


R is the radius of the head. R points to P and has the direction opposite to F_res_:
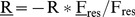
(4)


H can be substituted by
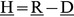
(5)With D being the orthogonal projection of R on M_res_:



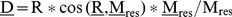
(6)The angle between R and M_res_ can be derived from their scalar product:
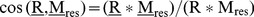
(7)


Applying (4), (6), (7), equation (5) becomes
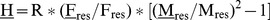
(8)


The friction coefficient µ is determined from (3), using the measured load vectors F_res_ and M_res_, the known head radius R, and the lever arm H, which is calculated by (8). Due to measuring errors, µ will be inaccurate if F_res_ or M_res_ is very small. Therefore, µ was only determined for F_res_ ≥20% BW *and* M_res_≥0.02% BWm.

In previous simulator tests, µ has mostly been determined in the sagittal plane. To compare our 3D-derived values with this data, we additionally calculated µ from the forces and moments measured in the sagittal, frontal, and horizontal planes as follows:
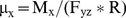
(9a)

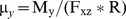
(9b)

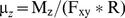
(9c)F_yz,_ F_xz_ and F_xy_ are the forces in the sagittal, frontal, and horizontal planes, respectively.

### Friction-induced power

In addition to the measured joint loads and the calculated friction coefficient µ, we determined the friction-induced power Q in the joint, which is caused by the friction force F_friction_. With v being the gliding speed between head and cup, Q is given by the simplified equation
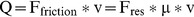
(10)


*Average* values of Q were calculated separately for the extension and flexion phases of the gait cycle:
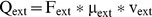
(11a)

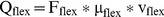
(11b)


The average power Q_aver_ over the *whole* gait cycle was then determined from
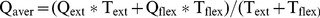
(11c)


Calculations were based on the intra-individually averaged load-time patterns of the single subjects. Before F_ext_, µ_ext_, F_flex_, and µ_flex_ were inserted in (11a, b), their time-dependent values were averaged arithmetically over the corresponding time periods T_ext_ and T_flex_. The speed v was determined from the flexion/extension range of the shank in the sagittal plane, the times of flexion and extension, and the radius of the prosthetic head. The data of 4–7 steps per subject were averaged. Because no gait analyses had been performed, the shank movement had to be determined from the patient videos. The Intra-observer variation of v and therefore Q was on average 1%, the inter-observer variability of four investigators was on average 2%.

## Results

Unless stated otherwise, the values reported here were taken from the time patterns of the average subject. The notation “X|Y” indicates a value of X at the instant of the first peak F_res1_ of the resultant force and a value of Y at the second peak F_res2_.

### Joint contact forces

**Figure 2:**
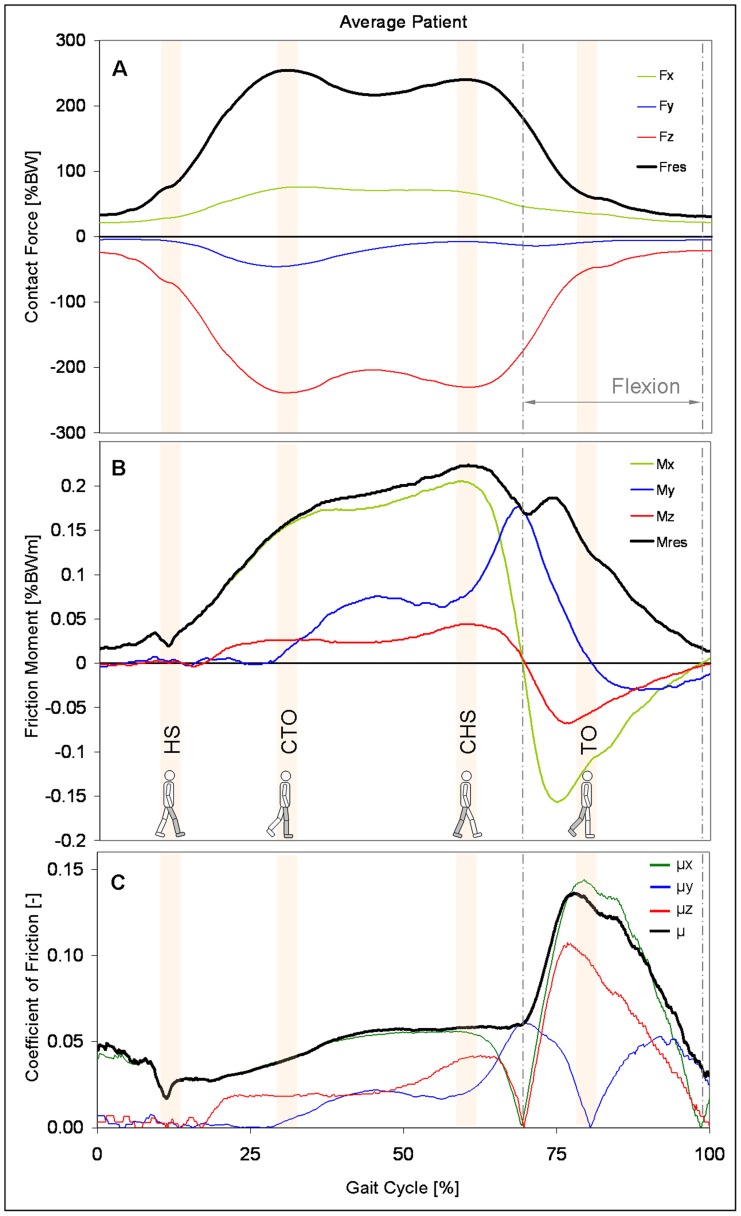
Time courses of contact force, friction moment, and coefficient of friction. Top: contact force F_res_ and its components. Middle: friction moment M_res_ and its components. Bottom: coefficients of friction.µ from 3D calculation; µ_x_ (sagittal plane), µ_y_ (frontal plane), and µ_z_ (horizontal plane) from simplified 2D calculations. The data presented are for an average subject during level walking at approximately 1 m/s. Vertical lines: borders of the flexion phase. The diagram starts before heel strike.

Figure 2A shows the time patterns of F_res_ and its components for the average subject during one walking cycle. F_res_ was nearly identical with -F_z_; the latter acts distally along the z-axis of the femur and compresses the hip joint. F_res_ had 2 peak values F_res1_|F_res2_. F_res1_ acted at about the time of contralateral toe off (CTO) and F_res2_ close to contralateral heel strike (CHS).

**Figure 3:**
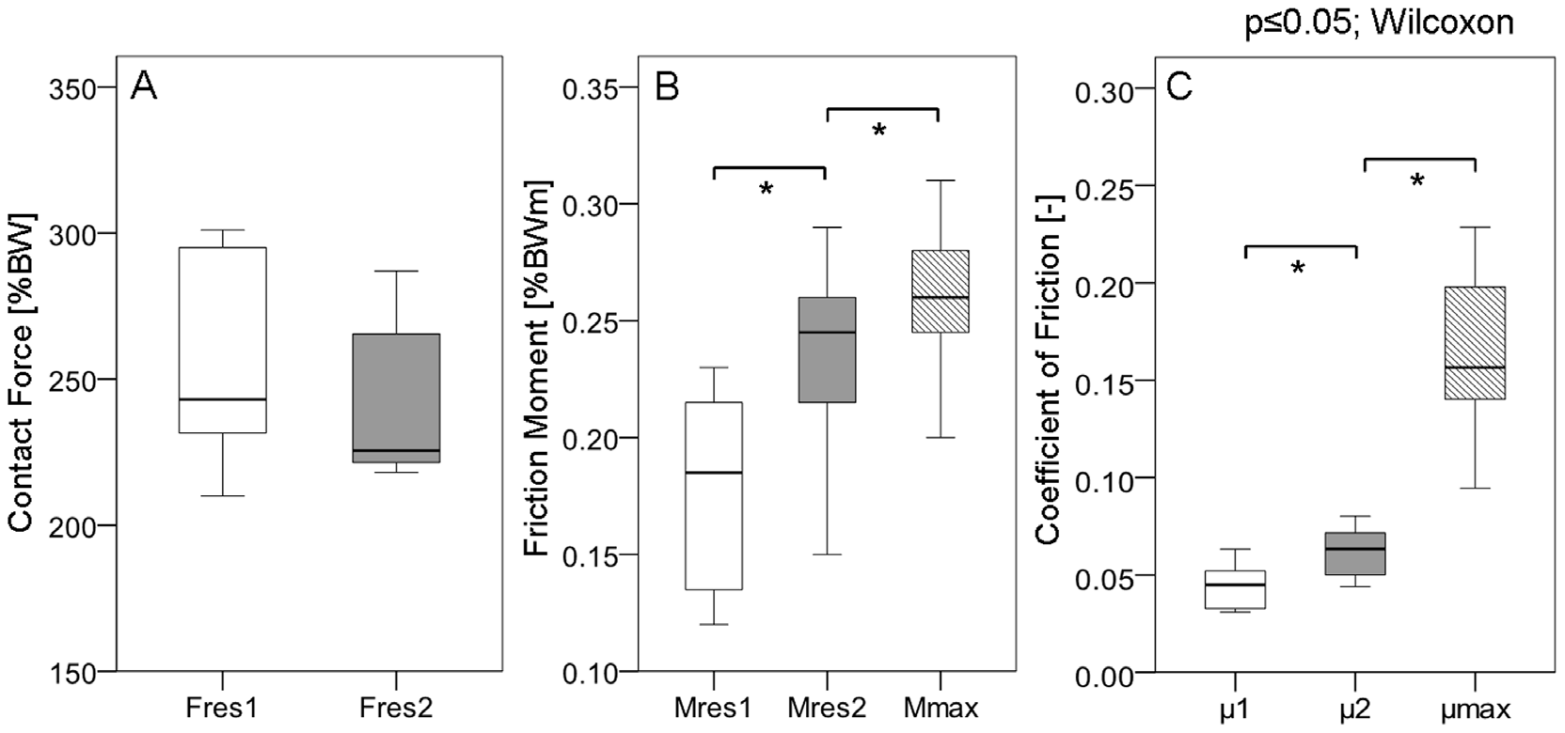
Peak values of contact force, friction moment and coefficient of friction. A: contact force F_res_, B: friction moment M_res_, C: coefficient of friction µ. Individual numerical mean values and ranges from 8 subjects. M_res1_|M_res2_, µ_1_|µ_2_ =  values at the instant of the force maxima F_res1_|F_res2_ (see Figure 2). M_max_, µ_max_  =  absolute maxima during a whole walking cycle.

Figure 3A contains individual numerical averages of the 8 subjects. For F_res1_|F_res2_ peak values of 248|233% BW at 31|57% of the gait cycle were determined. However, these peak forces varied widely inter-individually. F_res1_ ranged from 210% BW (subject H3) to 301% BW (H8), and F_res2_ from 218% BW (H3) to 287% BW (H8). In 6 subjects F_res1_ was higher than F_res2_, but in H2 and H3 F_res1_ was lower than F_res2_. The peak forces during the repeated trials of the same subject had standard deviations in the ranges of 7–14% BW (F_res1_) and 5–14% BW (F_res2_).

### Friction moments

Figure 2B shows the time courses of M_res_ and its components. M_res_ increased from heel strike (HS) to CHS and reached values of M_res1_|M_res2_  = 0.16|0.21% BWm. The maximum M_max_  = 0.22% BWm acted slightly later than the force maximum F_res2_. M_res_ climbed to a second, smaller peak of 0.19% BWm, which acted 15% of the cycle time after CHS, but before toe off of the ipsilateral leg (TO). During the intermediate minimum between CHS and TO, the joint rotation changed from extension to flexion. From HS to CHS, M_res_ was predominantly determined by component M_x_, which acts in the sagittal plane of movement. After that and until the end of the stance phase the absolute values of M_y_ in the frontal plane exceeded those of M_x_.

**Figure 4:**
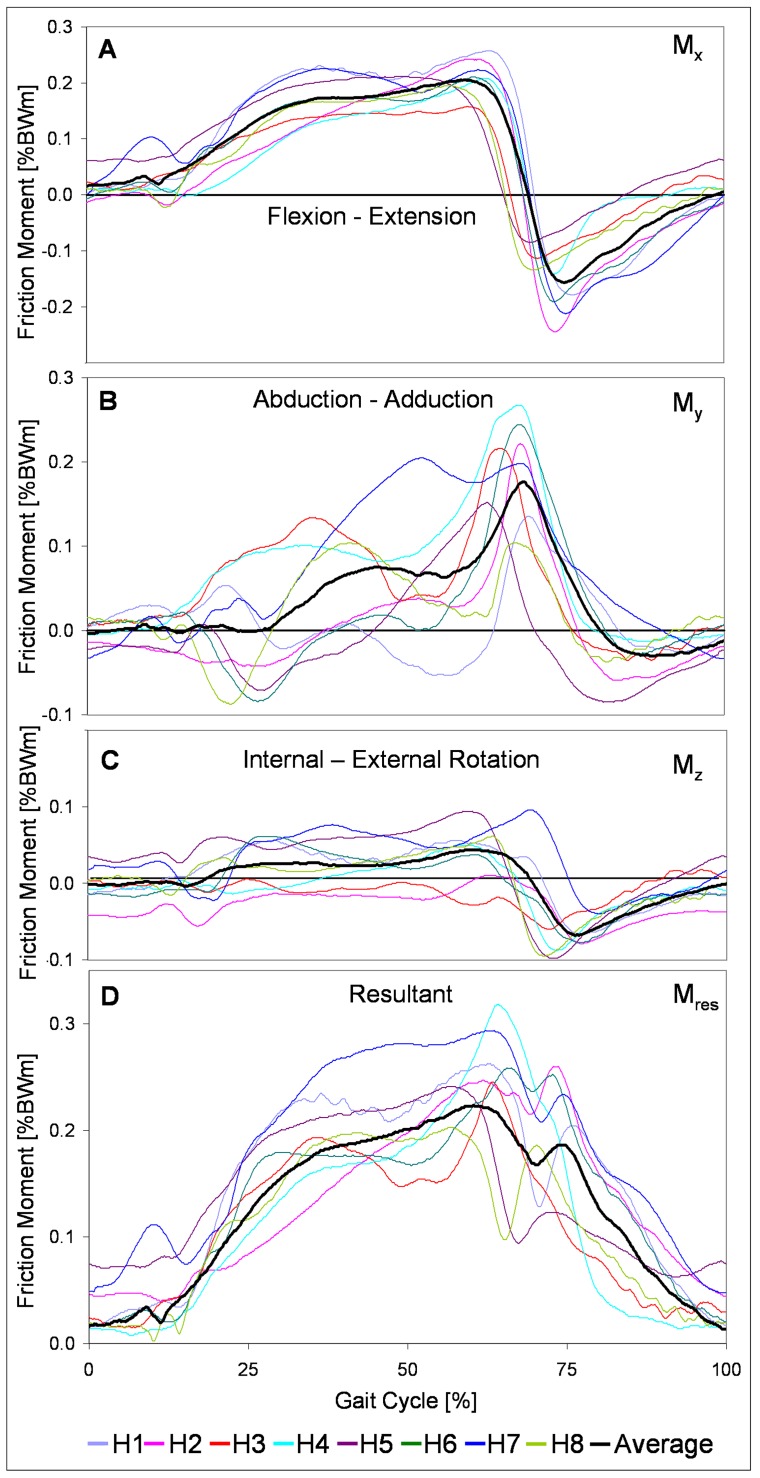
Components of friction moment. Components M_x_, M_y_, M_z_ around x, y, z axes (see Figure 1). Individually averaged patterns of subjects H1 to H8 (color), and average patterns of all subjects (black).

The patterns and magnitudes of M_x_ were relatively uniform for all 8 subjects (Figure 4A). On average the maximum of M_y_ had nearly the same magnitude as that of M_x_ (Figure 4B). The individual maxima of M_y_ (second peak value in subject H7) acted at very similar times. However, the variation of the individual maxima was much larger compared to M_x_. Especially during the first half of the stance phase the time courses of M_y_ individually varied greatly. On average the peak value of the moment M_z_ was about ¼ of that of M_x_ or M_y_ (Figure 4C). The individual time courses of M_z_ were similar, but the magnitudes varied considerably.

Figure 3B shows the averages and ranges of the peak values of M_res_ at the times of F_res1_|F_res2_. M_res1_|M_res2_ individually varied extremely with inter-trial standard deviations of 0.01 to 0.03% BW for both peak moments M_res1_ and M_res2_. M_res1_ ranged from 0.12% BWm (H2) to 0.23% BWm (H1), while M_res2_ lay between 0.15% BWm (H3) and 0.29% BWm (H7). In 7 subjects M_res_ increased between the times of F_res1_ and F_res2_ (Figure 4D), with peak values of M_max_ between 0.2% BWm (H8) and 0.32% BWm (H4); it decreased only in H3, but increased again after the time of F_res2_. In 5 subjects M_max_ occurred with a time delay after F_res2_ between 6% (H4) and 16% (H2) of the gait cycle; in 1 patient M_max_ occurred 2% before F_res2_ (H7), and in 2 subjects, no time delay was observed (H5 and H8). The individual inter-trial standard deviations of M_max_ lay between 0.01 and 0.03% BWm.

### Friction coefficients

At HS µ was the lowest with an average value of µ = 0.02 (Figure 2C) and then increased nearly linearly. The values at the times of F_res1_|F_res2_ were µ_1_|µ_2_ = 0.04|0.06. After the instant when the joint rotation changed from extension to flexion, µ rose dramatically and reached its maximum µ_max_  = 0.14, shortly before TO.

The individual numerical values of µ at the instants of F_res1_|F_res2_ varied by a factor of 2 (Figure 3B); µ_1_ had values between 0.03 (lowest in H2, H4, H8) and 0.06 (highest in H3); µ_2_ varied between 0.04 (H3, H8) and 0.08 (H7). The maxima µ_max_, which occurred shortly before TO, varied more, with values between 0.09 (H8) and 0.23 (H2).

The average patterns of the coefficients µ_x_, µ_y_, µ_z_, calculated by the two-dimensional approaches, changed throughout the whole gait cycle (Figure 2C). µ_x_ in the main plane of movement corresponded well to µ (3D) throughout most of the loading cycle. However, shortly before and after joint rotation changed from extension to flexion, µ_x_ fell to zero. During the flexion phase, especially at its end, µ_x_ also deviated from µ. A temporary decline similar to µ_x_ was also observed for µ_z_ in the plane of femur rotation, when joint movement changed from extension to flexion. At the same time µ_y_ in the abduction-adduction plane reached a maximum.

### Friction-induced power

**Figure 5:**
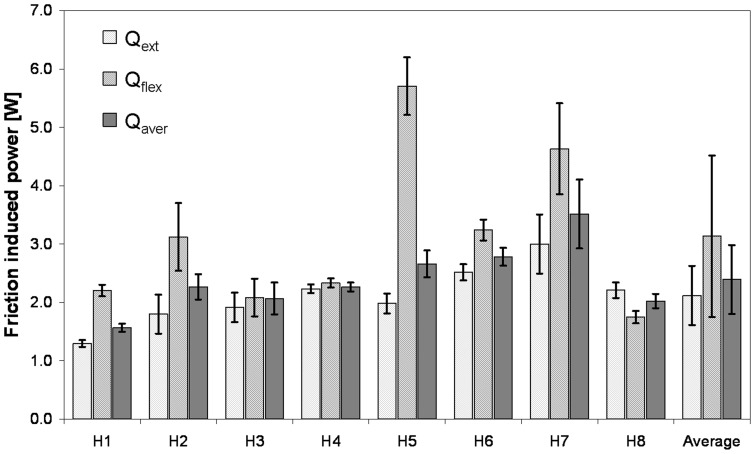
Average friction-induced power Q during flexion and extension phases and whole walking cycle. Q_ext_  = Q during extension phase; Q_flex_  = Q during flexion phase; Q_aver_  = Q during whole step; Individual values of subjects H1 to H8 and averages (right columns).

With Q_flex_  = 5.0 W, the highest friction-induced power was observed in subjects H5 and H7 during the flexion phase (Figure 5), although F_res_ and M_res_ were very small (Figure 2A) during most of this period. In 7 of the 8 subjects, Q_flex_ was higher than Q_ext_, which was mainly due to the higher values of µ and v during flexion (equation 11a, b). The individual differences between Q_flex_ and Q_ext_ varied considerably (Figure 5). The greatest difference was calculated for H5, in which Q_Flex_ was 2.9 times higher than Q_ext_. The smallest difference was 4%, observed in H4. In H8, Q_flex_ was 21% lower than Q_ext_. The inter-individual average power throughout the whole cycle was Q_aver_  = 2.3 W, with a range between 1.4 W (H1) and 3.8 W (H7). The average sliding speed during flexion was 2.2 times higher than during extension (Table 1), with individual values between 1.5 (H8) and 4.5 (H5).

## Discussion

This study reports for the first time on the assessment of *in vivo* friction in artificial hip joints during walking. The *in vivo* measured friction moment, at 3 month post-operative, increased during every gait cycle and as a consequence the coefficient of friction.

### Forces and friction moments

Different *in vitro* test conditions were applied by others when investigating friction in hip joint prostheses. Several studies investigated friction by moving the joint in one plane like a pendulum [18], [19], [29], [30], simulating flexion/extension, and neglected movement around the other 2 axes. Our results show that these test conditions may be much too simplified. In reality the abduction-adduction moment M_y_ rises to nearly the same peak value (0.18% BWm) as the flexion-extension moment M_x_ (0.2% BWm). Additionally, the joint contact force is not sinusoidal. It may affect the re-formation of a lubricating film during the swing phase when applying a sinus-load.

The resultant moment M_res_ shows a different time-course than the resultant force F_res_ (Figure 2A, B). Although the second force maximum is slightly lower than the first one, the moment is much higher at the instant of the second force peak. This is because friction continuously increases during the extension phase of walking (Figure 2C). The additional peak in the moment curve, shortly before TO, is caused by the sharp increase of the moments M_x_ and M_z_ when the hip begins to flex (see below). This finding stands in contrast to *in vitro* findings [18], [30], which are based on movements in only one plane. In these studies, the moment showed a plateau phase.

Micro-separation during the swing phase, as reported by others [31], [32], can alter the lubrication conditions in the joint. This effect was never observed in our subjects, investigated now and in the past. Otherwise fast changes in the directions of F_res_ or one of its components would have been observed.

### Coefficient of friction

The coefficient µ increases by 46% from HS to the instant when the joint starts to flex (Figure 2C). Directly after the change of joint movement from extension to flexion, µ rose sharply in all subjects and reached its maximum at about TO, when the resultant force has markedly been fallen already. This effect has not been described previously in such detail.

It must be assumed that the synovia is squeezed out by the high forces during the extension. *In vitro* studies reported that µ increases when the sliding properties change from lubricated to dry [17], [21], [22], [35]. Furthermore numerical studies with hard/hard pairings have shown that the thickness of the fluid film changes in relation to the joint loading during the gait cycle [30], [34]. It was shown that the fluid film thickness decreases at the end of the swing phase, and therefore µ and wear rise, if the swing phase load is increased [30]. A higher swing phase load prevents or restrains the re-formation of the lubrication film, required for good lubrication during the subsequent stance phase. If this explanation also holds true for the investigated ceramic/polyethylene combination of head/cup, a higher swing phase load would let µ decrease less sharply after TO and thus raise the level at which µ starts at the next HS.

Moreover, the strong increase of µ when the joint starts to flex could possibly be caused by a breakdown of the fluid film at the temporary stop of the relative joint movement, similar to the *in vitro* observation during the first step after a rest [33].

Another factor influencing the time pattern of µ may be a changing size of the load bearing area between head and cup, perpendicular to the resultant force vector. This could be especially true if µ were dependent on the pressure magnitude. Both factors could not be investigated in this study.

In contrast to previous *in vitro* studies, the *in vivo* coefficient of friction was now determined for the 3D case. The obtained pattern of µ differs from the pattern of µ_x_ in the main movement plane of the femur. Both coefficients are nearly identical from HS to CHS and deviate by no more than 5%. However, during the flexion phase, the difference between both coefficients increased up to 9% at TO.

Studies with a simple pendulum test determined values of µ_x_ between 0.04 and 0.09 for a lubricated regime [18], [36], [37]. This compares well with our finding of µ_1_ = 0.04 and µ_2_ = 0.06 during the extension phase. However, µ_max_  = 0.14 at the instant of toe off was much higher in our study.

The peak values of F_res_ individually varied by 39%, but the peak values of µ differed by 246% (Figure 3C). The variance of µ is most likely due to individually different lubrication properties of the synovia fluid.

### Friction-induced power

The friction-induced temperature rise in ceramic/PE implants has been measured *in vivo* previously [10]. An estimated average friction-induced power of 0.79 W during walking was reported, which is much less than the average of 2.3 W determined in the current study. It may be that heat convection by the blood flow has been underestimated in the previous study. Other reasons for this discrepancy may be differences between the subjects investigated, and the small sizes of both cohorts. This assumption is supported by another result of the cited study, namely that the temperature increase, measured after 1 hour of walking, individually varied by a factor of nearly 3 after the body weight of all subjects had been standardized. A similarly large factor of 2.7 was observed for the friction-induced power Q_aver_, which decreased to 2.3 after normalizing the body weight.

The large individual differences of Q_aver_ are most likely caused by varying synovia properties, as already indicated by the variations of µ. Additionally, different running-in effects of the gliding partners may affect the friction-induced power. This running-in effect and the expected decrease of µ and Q_aver_ with increased postoperative time will be investigated in a future study.

In conclusion it was shown: The friction moment in the hip joint mainly occurred in the frontal and sagittal plane during walking. The resultant coefficient of friction increased nearly linearly during extension and increased drastically in the beginning of flexion with the maximum value approximately the ipsilateral toe off. This suggests that the synovia is squeezed out of the intra-articular joint space. Furthermore, the peak values of the coefficient of friction were always determined during the flexion phase. This indicates that the lubrication regime certainly changed into a dry phase at every step.

### Limitations of the study

There are some limitations to this study: the number of investigated subjects was small; they were younger than the majority of hip-replacement patients; and only one implant type was investigated at only one speed of walking and one time after implantation. However, the large individual variations of friction coefficient and generated power, as well as the changes of the friction coefficient throughout the gait cycle will probably not be much influenced qualitatively by age or materials. The effects of walking speed and postoperative time is currently investigated an additional study.
